# Knockdown of platinum-induced growth differentiation factor 15 abrogates p27-mediated tumor growth delay in the chemoresistant ovarian cancer model A2780cis

**DOI:** 10.1002/cam4.354

**Published:** 2014-12-10

**Authors:** Julia C Meier, Bernard Haendler, Henrik Seidel, Philip Groth, Robert Adams, Karl Ziegelbauer, Bertolt Kreft, Georg Beckmann, Anette Sommer, Charlotte Kopitz

**Affiliations:** 1 Global Drug Discovery, Bayer Pharma AGBerlin, Germany; 2 Free University of Berlin, Institute for BiologyBerlin, Germany; 3 Humboldt University Berlin, Institute for BiologyBerlin, Germany

**Keywords:** AKT, GDF15, linear model analysis, ovarian cancer, platinum resistance

## Abstract

Molecular mechanisms underlying the development of resistance to platinum-based treatment in patients with ovarian cancer remain poorly understood. This is mainly due to the lack of appropriate in vivo models allowing the identification of resistance-related factors. In this study, we used human whole-genome microarrays and linear model analysis to identify potential resistance-related genes by comparing the expression profiles of the parental human ovarian cancer model A2780 and its platinum-resistant variant A2780cis before and after carboplatin treatment in vivo. Growth differentiation factor 15 (GDF15) was identified as one of five potential resistance-related genes in the A2780cis tumor model. Although A2780-bearing mice showed a strong carboplatin-induced increase of GDF15 plasma levels, the basal higher GDF15 plasma levels of A2780cis-bearing mice showed no further increase after short-term or long-term carboplatin treatment. This correlated with a decreased DNA damage response, enhanced AKT survival signaling and abrogated cell cycle arrest in the carboplatin-treated A2780cis tumors. Furthermore, knockdown of GDF15 in A2780cis cells did not alter cell proliferation but enhanced cell migration and colony size in vitro. Interestingly, in vivo knockdown of GDF15 in the A2780cis model led to a basal-enhanced tumor growth, but increased sensitivity to carboplatin treatment as compared to the control-transduced A2780cis tumors. This was associated with larger necrotic areas, a lobular tumor structure and increased p53 and p16 expression of the carboplatin-treated shGDF15-A2780cis tumors. Furthermore, shRNA-mediated GDF15 knockdown abrogated p27 expression as compared to control-transduced A2780cis tumors. In conclusion, these data show that GDF15 may contribute to carboplatin resistance by suppressing tumor growth through p27. These data show that GDF15 might serve as a novel treatment target in women with platinum-resistant ovarian cancer.

## Introduction

Epithelial ovarian cancer remains the fifth leading cause of cancer death in women worldwide, despite a small decline of the mortality rate in the last 10 years.^1^–^3^ A 90% cure rate is observed in early-stage ovarian cancers, however, in advanced stages (III-IV), about 20–30% of patients never have a remission or will suffer from recurrent disease within 5 years.^4^ This is due to the lack of appropriate diagnostic markers for early-stage ovarian cancers and to the development of resistance against standard treatments, which include debulking surgery and platinum-based chemotherapy.^5^,^6^ Platinum-based compounds such as cisplatin and carboplatin exert their cytotoxic activity in proliferating cells mainly by formation of inter- and intrastrand DNA adducts which trigger ATM/ATR DNA damage response (DDR). Several DNA damage transduction pathways including the p53, the p38 MAPK and the AKT pathways are then activated, leading to cell cycle arrest and DNA repair or apoptosis.^7^,^8^ Beside decreased drug uptake and increased drug efflux, resistance mechanisms can occur due to modified DDR, increased DNA repair, deficient cell cycle arrest, or enhanced survival signaling.^9^,^10^

Growth differentiation factor 15 (GDF15), a stress-responsive cytokine, shows elevated levels in effusions of ovarian cancer patients and is associated with short overall survival and poor response to chemotherapy at first disease recurrence.^11^,^12^ As a member of the TGF-ß superfamily, GDF15 shares 25% sequence homology with bone morphogenetic proteins (BMPs) and is expressed as a dimeric pro-protein and secreted into the extracellular space.^13^ As GDF15 levels are increased through cellular stress signals such as inflammation, acute tissue injuries, and malignant progression, this protein may represent a potential biomarker for the diagnosis and progression monitoring of cancer, and also for the risk of heart failure.^14^–^17^ Furthermore, GDF15 is upregulated upon treatment with various anticancer agents including NSAIDs, PPAR*γ* ligands, doxorubicin, genistein, and carboplatin.^18^–^20^ Importantly, this factor has also been implicated in resistance to chemotherapeutic drugs in lung, colon, prostate, and breast cancer.^21^ The transcriptional induction of GDF15 through p53, SP1, EGR1 as well as PI3K/AKT and ERK1/2 signaling has been demonstrated in several cancer types,^22^,^23^ but very little is known about the molecular interaction network of GDF15 and its link to the development of chemoresistance in ovarian cancer. In contrast to established tumor cell lines in vitro, in vivo tumor models express a larger pool of factors potentially involved in chemoresistance, linked to their three-dimensional growth and the modulation of ECM components, macrophages, or endothelial cells during angiogenesis.

The aim of this study was the identification of platinum-regulated genes and pathways for a better understanding of the molecular basis of platinum resistance in the ovarian cancer model A2780cis in vivo.

In this study, we found GDF15 levels to be highly enhanced during carboplatin treatment in the platinum-sensitive ovarian cancer model A2780 but not in the platinum-resistant model A2780 in vivo. Furthermore, knockdown of GDF15 in A2780cis in vivo resulted in enhanced subcutaneous tumor growth in mice but increased sensitivity to carboplatin treatment. This was accompanied by altered tumor histology, a downregulation of p27^Kip1^ and stabilization of phospho-p53 during carboplatin treatment. In summary, we showed that GDF15 might serve as a novel treatment target in women with platinum-resistant ovarian cancer.

## Materials and Methods

### Cell lines and reagents

The human ovarian carcinoma cell line A2780 and its cisplatin-resistant variant A2780cis 24 were obtained from ECACC (European Collection of Cell Cultures), cultured in RPMI1640/10% FCS (fetal calf serum) as recommended by ECACC and authenticated by the DMSZ (German Collection of Microorganisms and Cell Cultures, Germany) through DNA (STR) profiling. Cisplatin and carboplatin were purchased from Sigma (Germany, Hamburg) and dissolved in DMSO for in vitro use or in 0.9% NaCl for in vivo administration. A2780cis cells were continuously cultivated in presence of 5 *μ*mol/L cisplatin.

### Proliferation assays

3 × 10^3^ cells/well were seeded in 96-well plates and treated 24 hours later with different concentrations of carboplatin or 0.1–0.5% DMSO (Sigma). 48 hours later, CellTiter-Glo® (Promega, Mannheim, Germany) was added and luminescence was measured. IC_50_ values were calculated.

### In vivo studies

Animal experiments were conducted in accordance with the German animal welfare law, approved by local authorities and in accordance with the ethical guidelines of Bayer AG. Female fox-chase SCID mice (CBd17/ctrl), 5–6 weeks old (Charles River Laboratories, Sulzfeld, Germany) were injected subcutaneously with 0.5 × 10^6^ A2780 or A2780cis cells in 100% BD Matrigel™ (BD Biosciences, Heidelberg, Germany), into the right flank. Body weight and tumor area (length × width) were assessed at least twice weekly. Tumors were allowed to grow up >20 mm^2^ (long-term carboplatin treatment) or >50 mm² (short-term carboplatin treatment) after which mice were randomly assigned to experimental groups. Intravenous (i.v.) tail vein injection of 75 mg/kg carboplatin (Q1Dx2) for the short-term study and 50 mg/kg carboplatin (Q4Dx) for the long-term study was started. Control mice received equal volumes and schedules of vehicle alone (0.9% NaCl). Twenty-four hours after last treatment (short-term study) or when tumors reached ≥150 mm² (long-term study), respectively, mice were sacrificed, tumors obtained, weighted, bisected, and either snap-frozen for RNA and protein analyses or preserved for histological analyses. Plasma was collected at defined time points during tumor growth (as indicated) via retrobulbar puncture and during sacrifice. Effects of treatment on tumor growth were assessed by determining time until tumor area reached ≥150 mm^2^.

### Microarray hybridization and quantitative real-time PCR

cRNAs of A2780 and A2780cis tumors (30 mg, n=4-5) were hybridized to human whole-genome Illumina HT-12v3 bead chips (ArrayExpress Accession no. E-MTAB-1858). More details are given in Fig. S1. cDNA synthesis was performed with SuperScript® (Invitrogen, Darmstadt, Germany) according to manufacturer's instructions and quantitative real-time polymerase chain reaction (qRT-PCR) was conducted with TaqMan® gene expression assays (Applied Biosystems, Darmstadt, Germany) for GDF15 (Hs00171132_m1), DRAM1 (Hs01014914_m1), SULF2 (Hs01016476_m1), P4HA2 (Hs00989996_m1), LMNA (Hs00153462_m1), cyclophilin (4326316E) using TaqMan Universal Mastermix (Applied Biosystems). Relative mRNA expression 2^−ΔΔCt^ was calculated based on ΔCt to cyclophilin and ΔΔCt to mean mRNA expression of vehicle-treated A2780 tumors.

### Histological analyses

5 *μ*m and 3 *μ*m sections of TissueTek-cryo-preserved and formalin-fixed paraffin-embedded (FFPE) tumors, respectively, were used for histological analyses. Hematoxylin and eosin staining (H&E, Sigma) was conducted on cryo and FFPE sections. Tumor grade was determined by an experienced pathologist in three tumors/group and 10 high-power fields each. TUNEL assay (Roche, Mannheim, Germany) was performed on cryo sections according to the manufacturer's instructions. For Ki-67 staining, cryo sections were fixed with formalin, peroxidase blocked (Dako, Hamburg, Germany), incubated for 1 hour at room temperature (RT) with biotinylated mouse anti-human Ki-67 (Dako), and visualized with streptavidin-peroxidase (Dako) and diaminobenzidine (Dako).

### Biochemical assays

Xenogenic tumors and in vitro cultivated A2780 or A2780cis cells were lysed in lysis buffer (Meso Scale). For immunoblot analysis, 20–40 ng protein were separated on 10% sodiumdodecyl sulphate polyacrylamide gel electrophoresis (SDS-PAGE; Invitrogen), transferred onto nitrocellulose membranes (Invitrogen), blocked, and incubated with primary antibody overnight at 4°C. The following primary rabbit anti-human antibodies were used: AKT (4685), phospho-AKT (Ser473) (4060), p53 (7F5) (9284), phospho-p53 (Ser15) (2527), p21^Waf1/Cip1^ (12D) (2947), cyclin D1 (Santa Cruz, sc-718), Bcl-2 (2870), PCNA (2586), and GAPDH (2118), all purchased from Cell Signaling unless indicated otherwise. After washing, membranes were incubated with secondary anti-rabbit AlexaFluor680 antibody (Abcam, Cambridge, UK) and visualized using Odyssey infrared imaging system (LI-COR). GDF15 levels in cell supernatants, murine plasma, and tumor lysates were quantified with human GDF15 ELISA (DGD150; R&D Systems, Wiesbaden, Germany), whereby cell supernatants were collected after starving cells for 4 hours in RPMI1640 with reduced FCS (0.5% v/v). For the Proteome Profiler Arrays (R&D Systems) 300 *μ*g protein and for the Apoptosis Whole Cell Lysate kit (Meso Scale, Rockville, MD) 80 *μ*g protein were used from whole cell lysate of vehicle or carboplatin short-term-treated A2780 and A2780cis tumors. Processing was according to the manufacturer's instructions.

### shRNA-mediated knockdown and colony formation assay

4 × 10^5^ A2780cis cells/well were seeded in 12-well plates. Medium was removed the following day and lentiviral particles including vector backbone with nontargeting shRNA (TRC1, Sigma, SHC002) or shRNA targeting GDF15 (Sigma, TRCN0000058389 [shGDF15-1], TRCN0000058390 [shGDF15-2], TRCN0000058391 [shGDF15-3], TRCN000005892 [shGDF15-4]) and a puromycin selection site were applied at a MOI of 7 (multiplicity of infection) to A2780cis cells, followed by 6 hours incubation at 37°C_._ After medium change, shGDF15-(1-4)-transduced cells were selected and cultivated in RPMI1640/10% FCS supplemented with 1 *μ*g/mL puromycin (Sigma). For colony formation assays, 500 shGDF15- or shTRC1-A2780cis cells were plated with 300 000 A2780cis feeder cells and incubated at 37°C. Five, ten, twelve, 14, 18, and 26 days posttransduction, colonies were fixed with glutaraldehyde and stained with Crystal Violet (Sigma) for 30 min (RT), washed and air-dried. For quantification of the colonies, crystal violet was dissolved in 10% acidic acid and OD 595 nm was measured. The colony size and morphology was qualitatively analyzed by microscopy.

### Migration assay

A2780, shGDF15-, TRC1-, and nontransduced A2780cis cells were starved in RPMI1640 with reduced FCS (0.5% v/v) alone or 50 *μ*M carboplatin for 24 hours. The following day, 1 × 10^**5**^ cells in RPMI1640 and 0.5% or 10% FCS were added to the upper chamber of transwell plates (8 *μ*mol/L pore membrane, BD Falcon). Twenty-four hours later, the number of migrated cells was determined by Calcein AM (BD Biosciences) fluorescence.

### Statistical analyses

In vivo experiments were analyzed using One-way-analysis of variance (ANOVA) followed by Bonferroni correction and/or Kaplan–Meier survival analysis of tumor area with GraphPad Prism 6 software. Illumina expression data were analyzed with (1) linear model analysis (Genedata Analyst 7.4.) with false discovery rate (FDR) <0.1 and *P* < 0.0001 and (2) One-way-ANOVA with *P* < 0.01 as significant cut off. Pathway enrichment analysis was performed with Pathway studio® 9 (Ariadne Genomics, Rockville, MD) at *P* < 0.00001 and Jaccard distance <0.01. Statistical significance is displayed as *P < 0.05, **P < 0.01, *** *P* < 0.001, ****P < 0.0001). Sample sizes (*n*) are given in the results part.

## Results

### Decreased apoptosis induction during carboplatin short-term treatment in A2780cis compared to A2780 tumor xenografts

**Figure 1:**
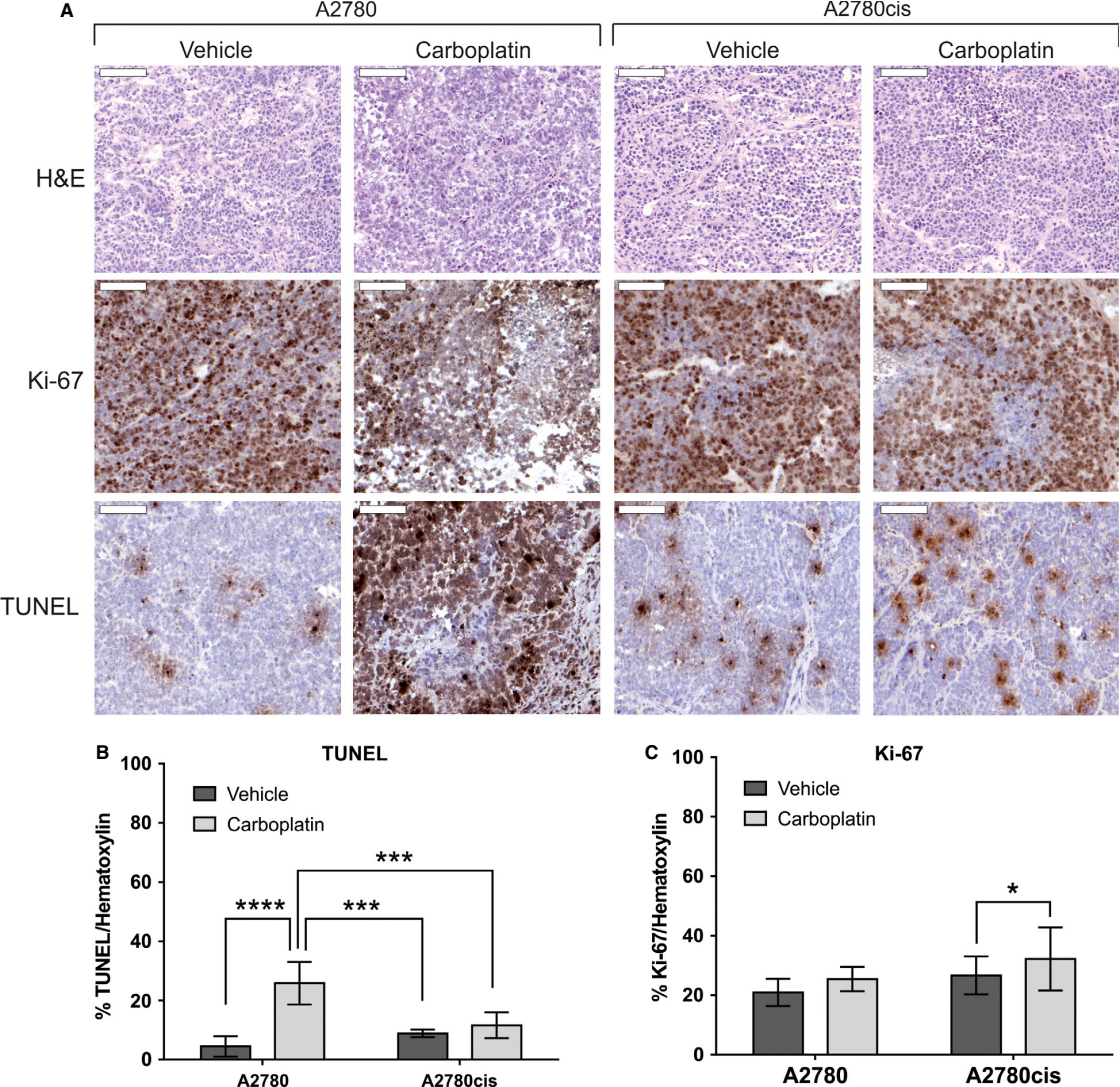
Proliferation and apoptosis in A2780 and A2780cis tumors after carboplatin short-term treatment. (A) Representative cryo sections after H&E, Ki-67 and TUNEL staining (*n* = 5/group). (B, C) Quantification of Ki-67 and TUNEL staining per hematoxylin-stained area in 10 high-power fields of three tumor specimens with ImageJ analysis. Statistical significance was determined with One-Way-ANOVA and Bonferroni post-hoc test, *P < 0.05, ***P < 0.001,****P < 0.000.

The aim of this study was to evaluate potential resistance-related factors that contribute not only to acquired resistance mechanisms, but are also involved in the initial response to platinum-based chemotherapy. Therefore, the human ovarian cancer cell lines A2780 and its cisplatin-resistant variant A2780cis were used as model system. To test whether A2780cis cells were also cross-resistant to the cisplatin analog carboplatin, we treated A2780 and A2780cis cells with various carboplatin concentrations and determined the IC50 concentrations in vitro. A2780cis showed a 30-fold decreased sensitivity (IC_50_ 1.1 × 10^−4^ mol/L) to carboplatin as compared to A2780 (IC_50_ 3.8 × 10^−6^ mol/L) (data not shown). Next, A2780 and A2780cis cells were subcutaneously transplanted onto SCID mice and 75 mg/kg carboplatin treatment was given for two consecutive days (short-term treatment). Following treatment, increased apoptosis in A2780 tumors was seen (5.7-fold), while no similar effect was observed in A2780cis tumors (1.2-fold), as evidenced by TUNEL staining (Fig.1A). Moreover, quantification of the TUNEL-positive tumor areas revealed a significantly higher basal apoptosis rate (twofold) in vehicle-treated A2780cis in contrast to A2780 tumors (Fig.1A and C). However, carboplatin treatment did not affect the number of cells staining positively for the proliferation marker Ki-67 in A2780 tumors. Interestingly, compared to vehicle treatment, slight but significant increase (1.2-fold) of Ki-67-positive cells was observed in A2780cis tumors following carboplatin treatment (Fig.1A and B).

### Decreased DDR and p53 phosphorylation but enhanced basal AKT activation in short-term carboplatin-treated A2780cis tumors

**Figure 2:**
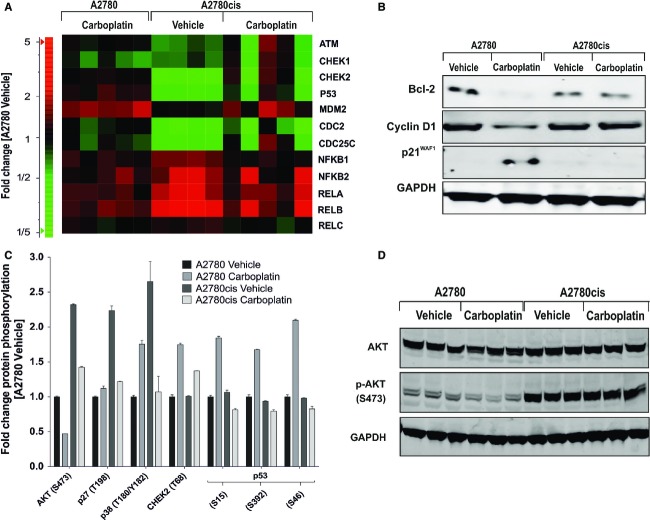
Analysis of genes involved in DNA damage response, cell cycle and survival signaling in A2780 and A2780cis tumors after carboplatin short-term treatment. (A) RNA expression analysis of DNA damage response and NF-*κ*B signaling assessed from Illumina bead chip array. (B) Bcl-2, cyclin D1 and p21^Waf1/Cip1^ protein levels. Tumors (*n* = 3) were pooled and subjected to western blot analysis. (C) Protein phosphorylation levels determined by Proteome Profiler Array. Significant differential protein phosphorylation was determined by comparison of all groups with Two-Way-ANOVA (*P* < 0.001). (D) Validation of Proteome Profiler Array data using phospho/total AKT western blot analysis.

Based on the result that short-term carboplatin treatment in A2780cis tumors showed a decreased apoptosis response, we asked which resistance-related factors might contribute to the phenotype in these tumors. We therefore performed a whole-genome gene expression analysis of short-term vehicle- and carboplatin-treated A2780 and A2780cis tumors on Illumina HT-12v3 arrays and examined the differential expression of genes involved in DDR, cell cycle control and cell survival (Fig.2A). When compared to vehicle-treated A2780 tumors, the vehicle-treated A2780cis tumors showed a lower RNA expression level of the DNA damage sensor ATM, of the downstream checkpoint kinases CHEK1 and CHEK2, and of p53. This was reversed by short-term carboplatin treatment in only two of five samples. For the p53 inhibitor MDM2, no difference in expression between vehicle- and carboplatin-treated A2780 and A2780cis tumors could be observed. Several components of the NF-*κ*B complex, with the exception of RelC, were expressed at higher levels in A2780cis compared to A2780 tumors and only slightly downregulated after carboplatin treatment in A2780cis tumors. Additionally, slightly reduced RNA levels of CHEK1 and CHEK2, and minimal upregulation of NF-*κ*B complex were observed following short-term carboplatin treatment in A2780 tumors as compared to vehicle-treated A2780 tumors (Fig.2A).

Further western blot analyses revealed a downregulation of the antiapoptotic factor Bcl-2 and of cyclin D1, while the cell cycle inhibitor p21^Waf1/Cip1^ was upregulated in A2780 tumors after short-term carboplatin treatment (Fig.2B). In contrast, no detectable effect on protein expression of Bcl-2, cyclin D1 or p21^Waf1/Cip1^ was seen in A2780cis tumors after short-term carboplatin treatment (Fig.2B). Differential protein phosphorylation was then investigated by a Proteome Profiler Array (Fig.2C). When compared to the sensitive counterparts, A2780cis tumors exhibited more than twofold higher phospho-AKT levels (S473) during vehicle treatment while carboplatin decreased these enhanced levels only slightly (1.6-fold). In contrast, A2780 tumors exhibited a 2.2-fold decrease of phospho-AKT levels as compared to vehicle treatment. Interestingly, phospho-p27^Kip1^ and phospho-p38*α* levels were significantly higher in A2780cis than in A2780 tumors during vehicle treatment. In this case, carboplatin treatment induced an upregulation of phospho-p38*α* levels in A2780 tumors, whereas strong downregulation was observed in A2780cis tumors. Moreover, p27^KIP1^ levels were 2.2-fold higher in vehicle-treated A2780cis than in vehicle-treated A2780 tumors, where they returned to the basal level following carboplatin treatment. In line with the microarray data, CHEK2 protein was weakly upregulated in A2780 and A2780cis tumors after short-term carboplatin treatment. The most prominent result of this analysis was a strong increase of phospho-p53 levels at all p53 phosphorylation sites (S15, S46, and S392) in short-term carboplatin-treated A2780 tumors, while no change was detected in A2780cis tumors. To further investigate these findings, total AKT, phospho-AKT were confirmed by western blot (Fig.2D). Whereas total AKT levels were unchanged in all four conditions, phospho-AKT (S473) levels were strongly enhanced in A2780cis compared to A2780 tumors. In A2780 tumors a slight phospho-AKT downregulation were seen after short-term carboplatin treatment.

### GDF15 is one of five resistance-related genes differentially expressed in short-term and long-term carboplatin-treated A2780 and A2780cis tumors

**Figure 3:**
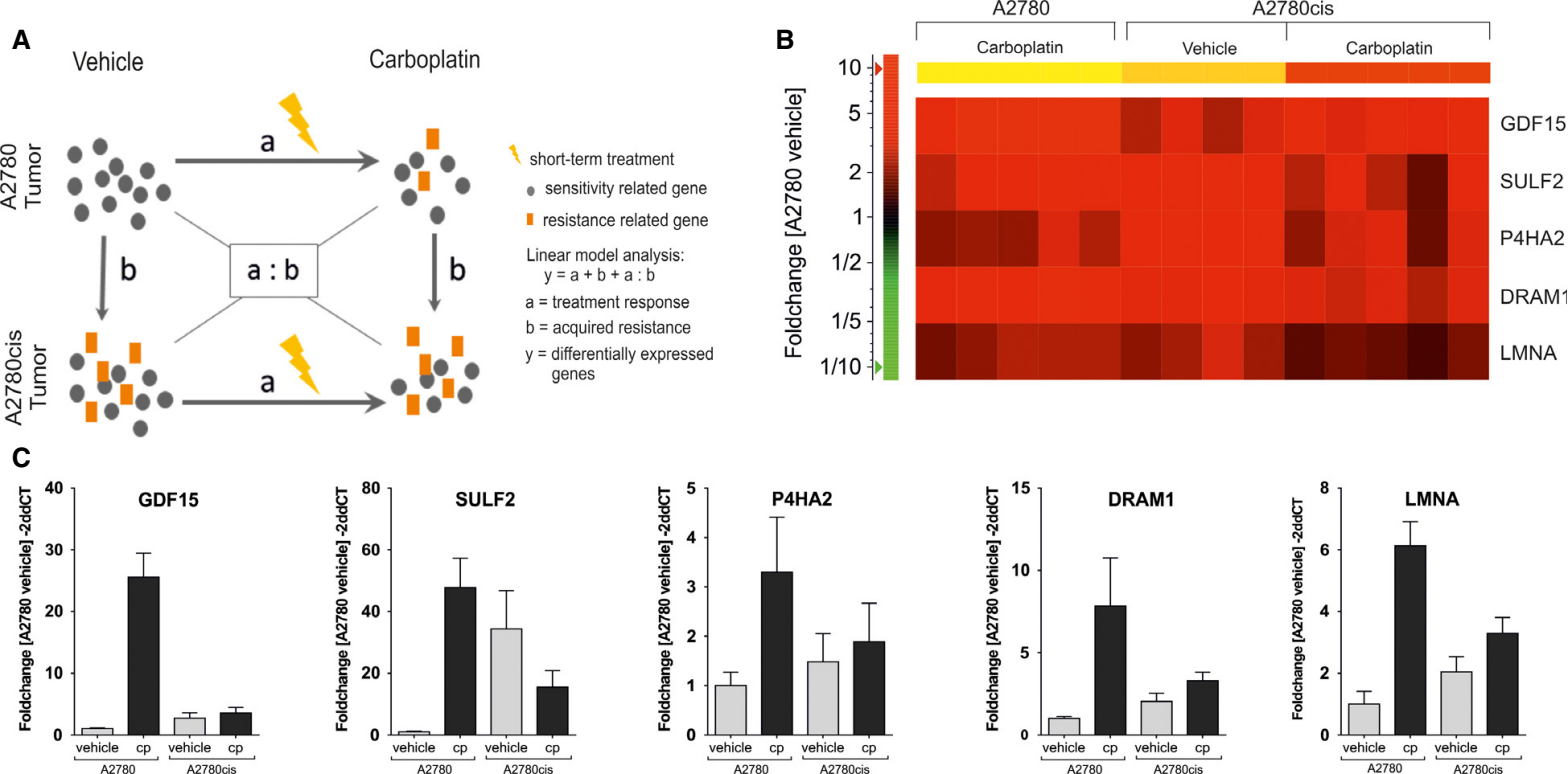
Differential expression of resistance-related genes in carboplatin short-term-treated A2780 and A2780cis tumors identified by linear model analysis. (A) Schematic view of linear model analysis for identification of resistance-related genes. (B) Heat map of differentially expressed genes determined by Illumina bead chip array. Genes showing RNA fold change >twofold in carboplatin-treated A2780 and vehicle-treated A2780cis tumors compared to vehicle-treated A2780 tumors. (C) qRT-PCR analysis of GDF15, DRAM1, SULF2, P4HA2, and LMNA expression.

Next, we hypothesized that resistance-related genes are initially induced through carboplatin treatment in A2780 tumors (initial response) and show higher basal expression level in vehicle-treated A2780cis tumors compared to vehicle-treated A2780 tumors (acquired resistance). Therefore, based on microarray data described above, linear model analysis (Expressionist™, Genedata, Munich, Germany) was performed to identify genes with significant interaction between treatment and resistance. The following experimental conditions were compared in the linear model: (1) Vehicle versus carboplatin treatment in A2780 and A2780cis tumors, respectively, and (2) A2780 versus A2780cis, tumors both during vehicle treatment (Fig.3A).

A total of 74 genes (FDR <0.1; *P* < 0.0001) with strong interaction between treatment and resistance were identified (Fig. S1). To define the role of the 74 differentially expressed genes, a pathway enrichment analysis was performed. All of them were predominantly involved in apoptosis (GO 0006915). Specifically, genome-wide *n*-way ANOVA of proapoptotic genes (GO 0006917) in this data set revealed that FAS expression was strongly upregulated in carboplatin-treated A2780 tumors, but not in vehicle- or carboplatin-treated A2780cis tumors (Fig. S2). Furthermore, based on a twofold RNA upregulation in vehicle-treated A2780cis as well as in carboplatin-treated A2780 tumors compared to vehicle-treated A2780 tumors, GDF15, DRAM1, SULF2, P4HA2, and LMNA were selected as potential resistance-related genes (Fig.3B). Quantitative RT-PCR confirmed that these five genes were upregulated in carboplatin-treated A2780 tumors and showed higher mRNA levels in vehicle-treated A2780cis tumors compared to vehicle-treated A2780 tumors (Fig.3C). In contrast, short-term carboplatin-treated A2780cis tumors showed no change in the mRNA levels of these genes (except SULF2) when compared to A2780cis vehicle-treated tumors. To provide additional evidence that the identified genes were involved in chemoresistance, we further analyzed the role of the cytokine GDF15 during A2780 and A2780cis tumor growth with and without carboplatin treatment. Based on previous studies which showed a correlation between high plasma GDF15 and poor treatment response in ovarian cancer patients,^11^ we measured the GDF15 plasma concentration following short- and long-term carboplatin treatment in A2780 and A2780cis tumor-bearing mice (Fig. S3). In accordance with microarray and qRT-PCR data, GDF15 levels were increased in plasma (14-fold) during carboplatin short-treatment in A2780 tumor-bearing mice compared to vehicle treatment, but only slightly (twofold) in A2780cis tumor-bearing mice. Additionally, basal GDF15 plasma levels were higher in A2780cis-bearing mice than in mice with A2780 tumors. During long-term treatment the GDF15 plasma levels in A2780cis-bearing mice increased rapidly with tumor growth, but were only slightly induced by long-term carboplatin treatment as compared to the GDF15 plasma levels in mice bearing the A2780 tumors. A similar carboplatin-induced GDF15 regulation could be detected in cell supernatants of DMSO-treated A2780 and A2780cis cells in vitro.

### GDF15 knockdown enhances colony size and migration of A2780cis cells in vitro

**Figure 4:**
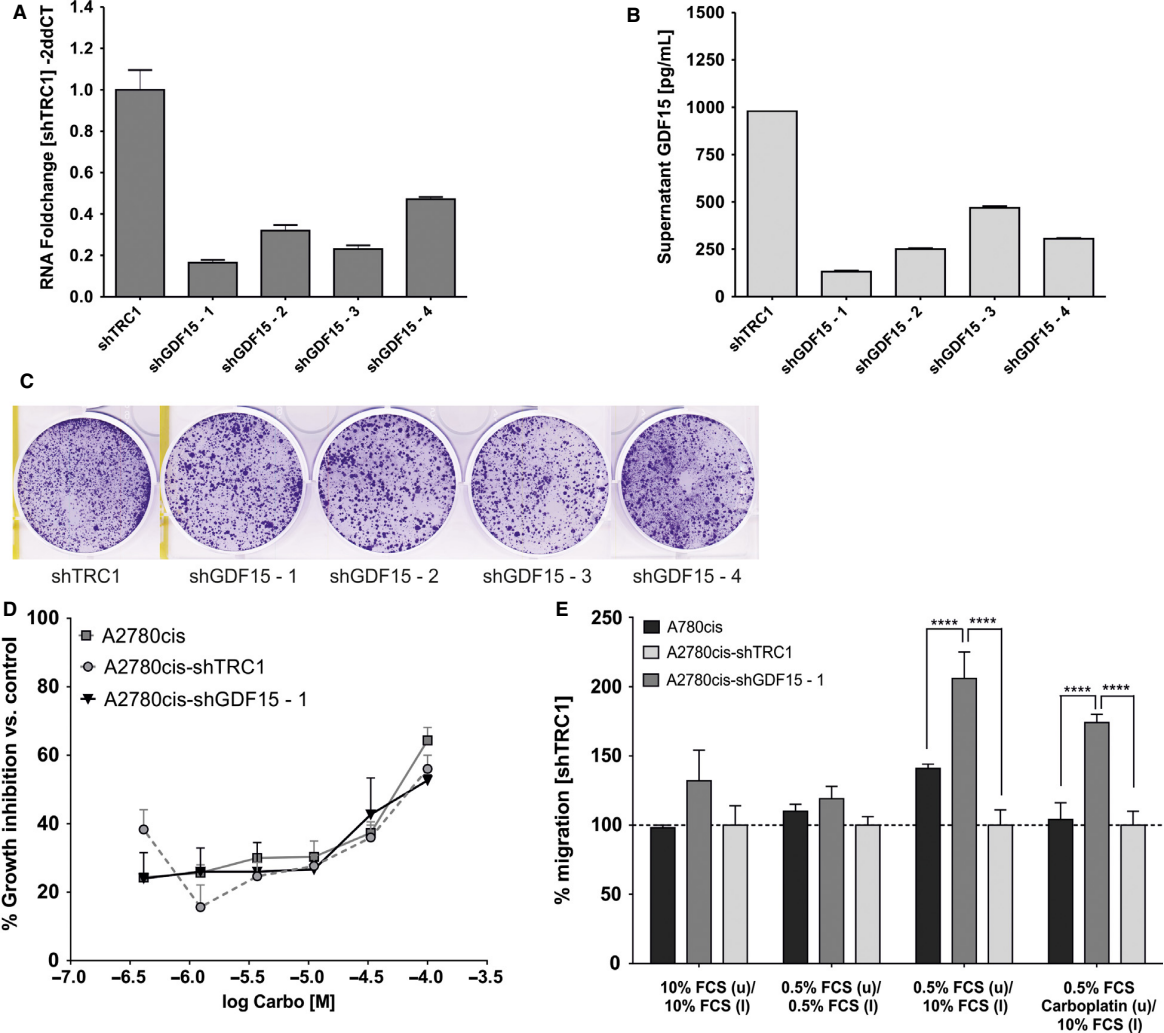
GDF15 knockdown in A2780cis cells in vitro. (A) qRT-PCR of GDF15 in shTRC1- and shGDF15-(1-4) stably transduced A2780cis cells. shGDF15-1 was selected for further analysis based on the strong GDF15 knockdown at mRNA (84%) and protein level (74%). (B) Analysis of supernatant of shTRC1- and shGDF15-(1-4)-transduced A2780cis cells. (C) Representative colony formation of shTRC1- and shGDF15-(1-4)-transduced A2780cis cells on day 14 posttransduction. (D) Growth curves of non-, TRC1- and shGDF15-transduced A2780cis cells during carboplatin treatment. (E) Migration of non- or 50 *μ*mol/L carboplatin-pretreated TRC1- non-, or shGDF15-transduced A2780cis cells with or without FCS gradient.

To assess whether GDF15 is causally involved in carboplatin resistance, shRNA-mediated knockdown of GDF15 was performed in A2780cis cells. In comparison to shTRC1-(control)-transduced A2780cis cells, over 50% reduction of GDF15 RNA levels was achieved in A2780cis with four different lentiviral shGDF15 transduction particles (Fig.4A). A comparable effect was observed for GDF15 protein levels in A2780cis cell culture supernatants (Fig.4B), whereas A2780cis-shGDF15-1 cells showed the highest GDF15 knockdown efficacy. In order to determine GDF15 knockdown effects on A2780cis cell proliferation, a colony formation assay was performed directly following transduction. Here, A2780cis cell colonies with silenced GDF15 had slightly larger diameter and higher distance between each single colony than shTRC1-transduced cells (Fig.4C). However, quantification of crystal violet staining revealed only a slightly higher number of cell colonies following shGDF15-(1-4) transduction as compared to shTRC1-transduced A2780cis (Table S1 and Fig. S4). Accordingly, IC_50_ determination of carboplatin in A2780cis-shGDF15-1 cells revealed no alteration of treatment response to carboplatin when compared to TRC1- or nontransduced A2780cis cells in vitro (Fig.4D). On the basis that nontransduced A2780cis cells showed a lower migration rate as compared to the nontransduced A2780 cells (Fig. S5), a transwell assay with shGDF15-A2780cis cells with or without a FCS gradient was performed. In the absence of FCS gradient, no difference in migration behavior was detected between TRC1-, non- or shGDF15-transduced A2780cis cells. Conversely, in a FCS gradient, GDF15 knockdown led to significantly enhanced numbers of migrating A2780cis cells compared to TRC1- or nontransduced A2780cis cells in absence and presence of carboplatin (Fig.4E).

### Knockdown of GDF15 in A2780cis accelerates tumor growth and increases carboplatin treatment response in vivo

**Figure 5:**
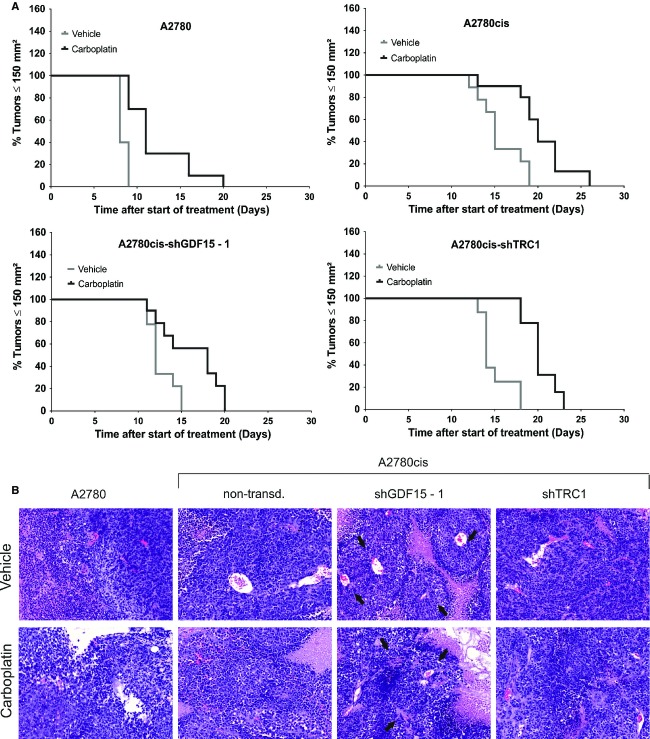
Growth of A2780, A2780cis, and shTRC1- and shGDF15-A2780cis tumors long-term treated with carboplatin. (A) Modified Kaplan–Meier survival curves. Tumor areas achieving ≥150 mm² were censored as zero events, indicating a “death event.” (B) Representative paraffin sections of H&E-stained A2780, A2780cis, shTRC1- and shGDF15-A2780cis tumors. Black arrows indicate the lobular arrangement of tumor cells in shGDF15-A2780cis tumors.

Next, we decided to find out whether the altered migratory behavior of A2780cis-shGDF15 cells in vitro would also result in modified tumor histology and tumor growth in vivo. A2780 cells, shGDF15-1-, TRC1- and nontransduced A2780cis cells were therefore transplanted subcutaneously onto SCID mice, treated with vehicle or carboplatin and the tumor growth was monitored until the predefined size of 150 mm² of tumor area. As expected, non- and TRC1-transduced A2780cis tumors showed a slower tumor growth and decreased response to carboplatin treatment as compared to A2780 tumors (Fig.5A). Interestingly, the shGDF15 knockdown in A2780cis tumors resulted in a significantly enhanced tumor growth (*P* < 0.0001) but also in a slightly better treatment response to carboplatin as compared to TRC1- and nontransduced A2780cis tumors (Table S2). In detail, A2780, nontransduced and shTRC1-A2780cis tumors showed a tumor growth inhibition between 33% and 37.5%, while GDF15 knockdown in A2780cis resulted in 50% tumor growth inhibition during vehicle treatment. This was associated with an overall change of tumor histology: H & E-stained A2780cis-shGDF15-1 tumors showed a viable peri-vascular tumor pattern at which the tumor cells were grouped radially around a central vein, which was further surrounded by large areas of necrotic tissue (Fig.5B). Semiquantitative analysis of tumor necrosis revealed a higher percentage of necrotic areas in A2780cis-shGDF15 compared to non- and TRC1-transduced A2780cis tumors while carboplatin treatment did not affect necrosis in the three A2780cis tumor models (Fig. S6). In summary, GDF15 knockdown in A2780cis tumors reversed the reduced growth rate and enhanced carboplatin insensitivity back to faster proliferating and carboplatin-sensitive A2780 tumor phenotype.

### Increased carboplatin response of shGDF15-A2780cis tumors is associated with reduced p27^Kip1^, but enhanced p53 phosphorylation

**Figure 6:**
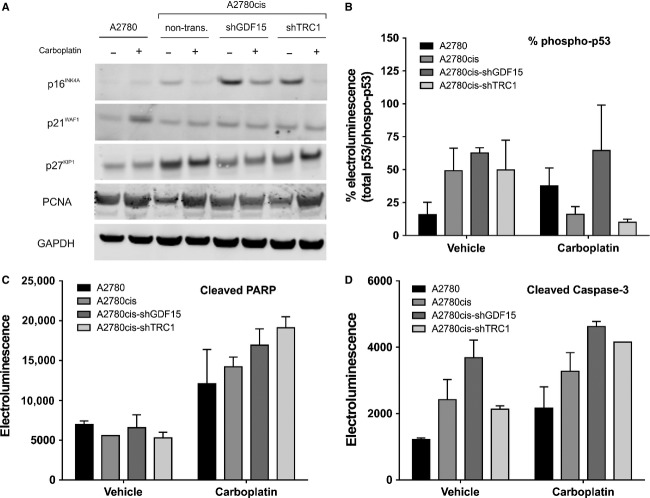
Apoptosis induction and cell cycle progression in A2780, A2780cis, shTRC1-, and shGDF15-A2780cis tumors after long-term carboplatin treatment. *n* = 3 tumors were pooled in each group. (A) Immunoblot of p16^INK^^4A^, p21^Waf1/Cip1^, p27^Kip1^, PCNA, and GAPDH. (B) Percent p53 activity measured by phospho-p53 normalized to total p53 electroluminescence. (C, D) Levels of cleaved PARP and cleaved caspase-3 measured by electroluminescence.

Finally, we investigated the potential causes of enhanced tumor growth but increased response to carboplatin treatment seen in A2780cis following GDF15 silencing. As expected, A2780 tumors showed p21^Waf1/Cip1^ upregulation following long-term carboplatin treatment, compared to vehicle-treated tumors (Fig.6A). Slightly higher basal p21^Waf1/Cip1^ levels were detected in shTRC1-, non- and shGDF15-transduced A2780cis tumors, which were not significantly affected by carboplatin treatment. Similarly, basal p27^Kip1^ were higher in shGDF15-, shTRC1- and nontransduced A2780cis tumors, but remained unaffected by carboplatin treatment. Interestingly, a slight downregulation of p27^Kip1^ was detected in vehicle- and carboplatin-treated shGDF15-A2780cis tumors as compared to shTRC1- and nontransduced A2780cis tumors. Even though high basal p16^INK4A^ levels could be detected in shGDF15-A2780cis tumors, the carboplatin treatment was able to downregulate these enhanced levels. However, a slightly enhanced p16^INK4A^ expression was also detected in shTRC1-A2780cis tumors. Furthermore, phosphorylation of p53 was much higher in all A2780cis samples, regardless of the GDF15 levels, compared to A2780 (Fig.6B). In contrast, a strong reduction of p53 phosphorylation was observed in TRC1- and nontransduced A2780cis tumors following carboplatin treatment, unless GDF15 expression was reduced. High induction of PARP cleavage was seen in A2780cis after carboplatin treatment. This was not affected by GDF15 knockdown (Fig.6C). Cleaved caspase-3 levels were higher in A2780cis than in A2780 cells, and slightly elevated after GDF15 knockdown. Additionally, treatment with carboplatin increased cleaved caspase-3 levels to similar levels in all A2780cis tumors, independently of the GDF15 levels (Fig.6D).

## Discussion

In this study we identified GDF15 as potential resistance-related factor acting by maintaining a low proliferative and undifferentiated tumor phenotype through upregulation of p27^Kip1^ during carboplatin treatment. Moreover, we postulate linear model analysis as a robust statistical test to identify carboplatin-regulated factors that might contribute to chemoresistance in ovarian cancer.

A total of 74 differentially expressed genes were identified in short-term carboplatin-treated A2780 and A2780cis tumors of which five, including GDF15, were further investigated. These five genes might contribute to chemoresistance in A2780cis, for example by enhanced DRAM1-dependent autophagy^25^ or enhanced collagen synthesis and nucleus membrane disassembly through P4HA2 and LMNA, respectively.^26^ In addition, Dai et al. showed SULF2 to be a tumor growth suppressor of myeloma in vivo,^27^ whereas other studies showed that enhanced SULF2 expression is correlated with poor prognosis of patients with primary multiple myeloma.^28^ These findings might indicate that A2780cis tumors undergo constitutive self-renewal but also exhibit a slow-growing phenotype. Furthermore, our data show that A2780cis tumors exhibited a higher basal GDF15 expression at RNA and protein level as compared to the carboplatin-sensitive A2780 tumors. This was associated with higher GDF15 plasma levels in the A2780cis tumor-bearing mice. Clinically, high GDF15 concentrations in effusions and tumor specimen of ovarian cancer patients correlate with poor overall survival and poor response to chemotherapy.^11^,^12^ Moreover, GDF15 overexpression has been described to be associated with resistance to various chemotherapeutic drugs, for example, cisplatin, docetaxel, and mitoxantrone in other cancer types.^29^–^32^ Our study shows for the first time that basal high GDF15 expression is associated with a carboplatin-resistant phenotype in ovarian cancer. Furthermore, GDF15 is a known p53-induced gene following treatment with NSAID, PPAR*γ* ligands and chemotoxic reagents like etoposid and doxorubicin resulting in a proapoptotic phenotype.^18^,^20^,^33^ This suggests, that the absence of GDF15 induction in the platinum-resistant tumor model A2780cis may indicate a decreased apoptosis response following carboplatin treatment.

The conversion of cytotoxic signals by platinum compounds in the nucleus is mainly facilitated by the ATM/ATR damage response pathway through downstream phosphorylation of p53.^34^ We found a basal mRNA downregulation of ATM and its downstream mediators CHEK2 and p53 in A2780cis tumors. This correlated with decreased p53 phosphorylation following carboplatin treatment. Thus, low ATM signaling in A2780cis tumors might circumvent p53 phosphorylation, thereby preventing cell cycle arrest or apoptosis after carboplatin exposure.^35^ We have also shown that p21^Waf1/Cip1^ protein expression was absent and Bcl-2 and cyclin D1 were unchanged in A2780cis tumors following carboplatin treatment. The p21^Waf1/Cip1^ inhibits cell cycle progression through binding to PCNA and cell cycle-dependent kinases (CDKs).^36^ Furthermore, a slight downregulation of phospho-AKT (Ser473) was detected in A2780 tumors while high basal phospho-AKT levels in A2780cis tumors remained unaffected following short-term carboplatin treatment. In contrast, Singh et al. described a cisplatin-mediated upregulation of phospho-AKT (Ser473) in A2780, but not in A2780cp cells in vitro.^37^ This suggests differential regulation of phospho-AKT in vitro and in vivo and/or different roles of cisplatin versus carboplatin in these cells. However, there is evidence that AKT activation plays a pivotal role in promoting resistance to platinum compounds and other chemotherapies in many cancer types, especially in ovarian cancer.^38^,^39^

Furthermore, we elucidated the pleiotropic functions of GDF15 in chemoresistance in ovarian tumors by generating a shRNA-mediated knockdown of GDF15 in A2780cis cells. In vitro, colony size and cell migration were enhanced in shGDF15-A2780cis cells, while proliferation and carboplatin-sensitivity were not affected. This is comparable to data shown by Liu et al., who found that GDF15 inhibits migration of prostate cancer cells and reduces cell adhesion but has no impact on tumor cell proliferation.^40^ A higher GDF15 level in the A2780cis cells might therefore suppress the migration of the A2780cis cells in vitro, while knockdown of GDF15 led to an enhanced migratory potential and altered colony formation of these cells. In contrast, other studies have shown an enhanced migratory and invasive potential of gastric cancer when GDF15 is overexpressed.^41^ This indicates that GDF15 may have different effects on migration and proliferation depending on the tumor cell type.

We found that subcutaneous transplantation of shGDF15-A2780cis cells into SCID mice led to a significant growth enhancement. Interestingly, this enhanced tumor growth resulted in an increased response to carboplatin treatment and modified tumor histology as compared to TRC1- or nontransduced A2780cis tumors. This was associated with a reduced p27^Kip1^ expression and higher DNA synthesis in shGDF15-A2780cis tumors. Thus, higher GDF15 levels in A2780cis tumors might decelerate tumor growth and therefore decrease the accessibility to replicating DNA for platinum compounds. Accordingly, it has been shown that GDF15 inhibits tumor growth of HCT-116, MCF-7, and PC-3 cells in nude mice and induces lower numbers of chemically or genetically induced gastrointestinal tumors in GDF15 transgenic mice.^42^,^43^ Similarly to p21^Waf1/Cip1^, the CDK inhibitor p27^Kip1^ controls the G1 to S phase transition and loss of p27^Kip1^ is frequent in many cancers.^44^,^45^ Thus, evidence exists that p27^Kip1^ and p53 affect quiescence in tumor cells when challenged with nutrient deficiencies, hypoxia, and genotoxic agents.^46^ We therefore propose that GDF15 suppresses tumor growth by the induction of p27^Kip1^ expression in A2780cis cells providing an escape mechanism from carboplatin treatment. Additionally, basal p16^INK4A^ was highly induced in shGDF15-A2780cis tumors. P16^INK4A^ exerts its tumor-suppressing functions for example by terminal cell differentiation following DNA damage.^47^ Furthermore, low p16^INK4A^ expression level is a predictor of poor treatment response in ovarian cancer patients.^48^ Thus, the altered tumor histology from an undifferentiated toward a structured, peri-vascular A2780cis tumor together with the enhanced p16^INK4A^ levels in GDF15-knockdown tumors suggests that GDF15 might represent a key factor in the suppression of epithelial cell differentiation, thereby allowing a constitutive self-renewable, stem-like tumor phenotype. Accordingly, Bonome et al. reported elevated GDF15 levels in serous ovarian tumors with low malignant potential and proposed an essential role of this factor in terminal differentiation and restricting cellular proliferation.^49^ This provides evidence that GDF15 might serve as a novel target for the sensitization of ovarian cancer patients to carboplatin treatment. However, the causal relationship of p16^INK4A^, p27^Kip1^, and GDF15 expression in chemoresistance remains to be elucidated.

In conclusion, we showed that high induction of GDF15 following carboplatin treatment distinguishes platinum-sensitive from platinum-resistant tumor specimen, while high basal levels of GDF15 before treatment may serve as a marker for chemoresponse in these tumor models. Moreover, like other TGF-*β* family members,^50^ GDF15 might serve as a proapoptotic factor in early-stage cancers, while promoting tumor progression in advanced disease. The role of GDF15and the amount of its secretion may be dependent on the cell type and progression in disease or treatment response. To translate these data into clinical relevance, more studies need to be undertaken, for example, in primary tumor specimens from ovarian cancer patients before, during and after carboplatin therapy. It is therefore of high medical interest to further elucidate GDF15-dependent signaling networks and the prognostic value of GDF15 in patients with ovarian cancer with regard to the development of carboplatin resistance.

## Supporting Information

**Figure S1.** Heat map of the 74 resistance-related genes that were identified by linear model analysis in short-term-treated A2780 and A2780cis tumors. Red color indicates RNA upregulation, green color downregulation. A2780 and A2780cis short-term carboplatin- and vehicle-treated bisected tumors (minimal size 30 mg, *n *= 4–5) were homogenized, RNA was isolated with RNeasy kit (Qiagen) and quality-controlled with RNA NanoChips (Agilent Technologies) at RIN ≥ 8–10 (RNA integrity number) according to the manufacturer's instructions. cDNA and cRNA were synthesized with the Ambion® Illumina TotalPrep RNA Amplification Kit (Invitrogen), hybridized to human whole-genome Illumina HT-12v3 bead chips (ArrayExpress: E-MTAB-1858) and mRNA expression was analyzed with Analyst software7.4 (Genedata). For qRT-PCR, RNA was reverse-transcribed into cDNA (SuperScript®, Invitrogen) according to the manufacturer's instructions.

**Figure S2.** Heat map of proapoptotic genes in short-term vehicle- or carboplatin-treated A2780 and A2780cis tumors determined by human whole-genome Illumina bead chip array. Red color indicates RNA upregulation, whereas green color shows downregulated genes. A One-way ANOVA of proapoptotic (GO ID: 0006917) genes was performed taking into account the fixed factors: cell type specification and treatment. A value of *P* < 0.001 was considered significant (effect factor: cell type specification).

**Figure S3.** Determination of GDF15 plasma levels in A2780 und A2780cis-bearing mice following short- and long-term carboplatin treatment. (A) GDF15 levels were increased 14-fold in plasma after carboplatin short-term treatment in A2780 tumor-bearing mice compared to vehicle treatment, while A2780cis tumors showed only a twofold increase. The basal GDF15 plasma levels were higher in A2780cis-bearing mice than in those with A2780 tumors. (B) GDF15 protein levels in cell supernatants of DMSO-treated A2780 and A2780cis cells in vitro. After carboplatin treatment, GDF15 was slightly more increased in the supernatant of A2780 (2.8-fold) cells than in A2780cis cells compared to DMSO-treatment (twofold). (C, D) GDF15 levels during continuous carboplatin exposure in subcutaneous A2780- and A2780cis tumors in vivo. Basal GDF15 plasma levels in A2780-bearing mice increased significantly upon long-term carboplatin treatment, reaching a peak (9.3-fold) around day 11 posttreatment start. After day 11, the plasma GDF15 levels decreased slightly (twofold) despite continuous carboplatin treatment. The GDF15 plasma levels in A2780cis-bearing mice increased rapidly with tumor growth, but were only slightly (1.4-fold at day 11 after start of treatment) induced by long-term carboplatin treatment.

**Figure S4.** Colony size and count after shRNA-mediated knockdown of GDF15 in A2780cis cells determined by digital counting with ImageJ Analysis Software in vitro. *n* = 3 crystal violet stained colony formation assays (14 days posttransduction, 5000 cells per 6 well (6 wellplate) were seeded and transduced) were analyzed and statistically compared by n-WAY ANOVA. ***P* < 0.01, ****P* < 0.001.

**Figure S5.** Migration of A2780 and A2780cis cells in a FCS gradient from medium supplemented with only 0.5% FCS ([u] upper chamber to medium containing 10% FCS [l] lower chamber) with or without 50 *µ*MOL/l carboplatin pretreatment for 24 hours. Migration was compared to a saturated FCS gradient with normal growth media containing 10% FCS. *n* = 3 transwell assays were performed for each condition.

**Figure S6.** Grading of the necrosis degree in subcutaneous long-term carboplatin-treated A2780 and A2780cis tumors, and TRC1- and shGDF15-A2780cis tumors. 10 high-power fields of two H&E sections of each tumor model treated with vehicle or carboplatin were graded from 1 to 5 with 1, minimal; 2, slight; 3, moderate; 4, massive; and 5, extreme.

**Table S1.** Quantity of crystal violet-stained A2780cis cell colonies transduced with shTRC1- or shGDF15 lentiviral particles.

**Table S2.** Statistical calculation of the median time of subcutaneous A2780 tumors and non-, shTRC1-, and shGDF15-A2780cis tumor growth (equivalent to “median survival” in Kaplan–Meier curve survival analysis) until tumor area reached the defined tumor size ≥150 mm² during vehicle or carboplatin treatment.
