# Supplementary figures and images for: Knockdown of platinum-induced growth differentiation factor 15 abrogates p27-mediated tumor growth delay in the chemoresistant ovarian cancer model A2780cis
Source: Cancer Med. 2014 Dec 10;4(2):253–67. doi: 10.1002/cam4.354 (PMC4329009; doi:10.1002/cam4.354)

*
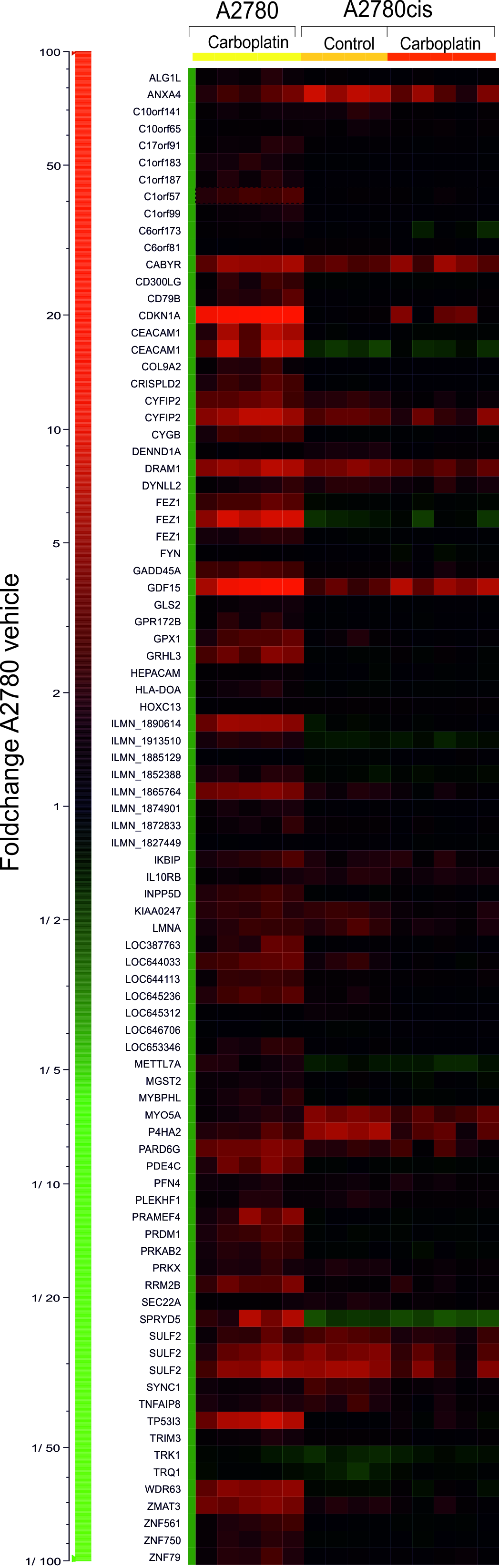
*

Supplement: Supplementary file 1 [file cam40004-0253-sd1.docx]

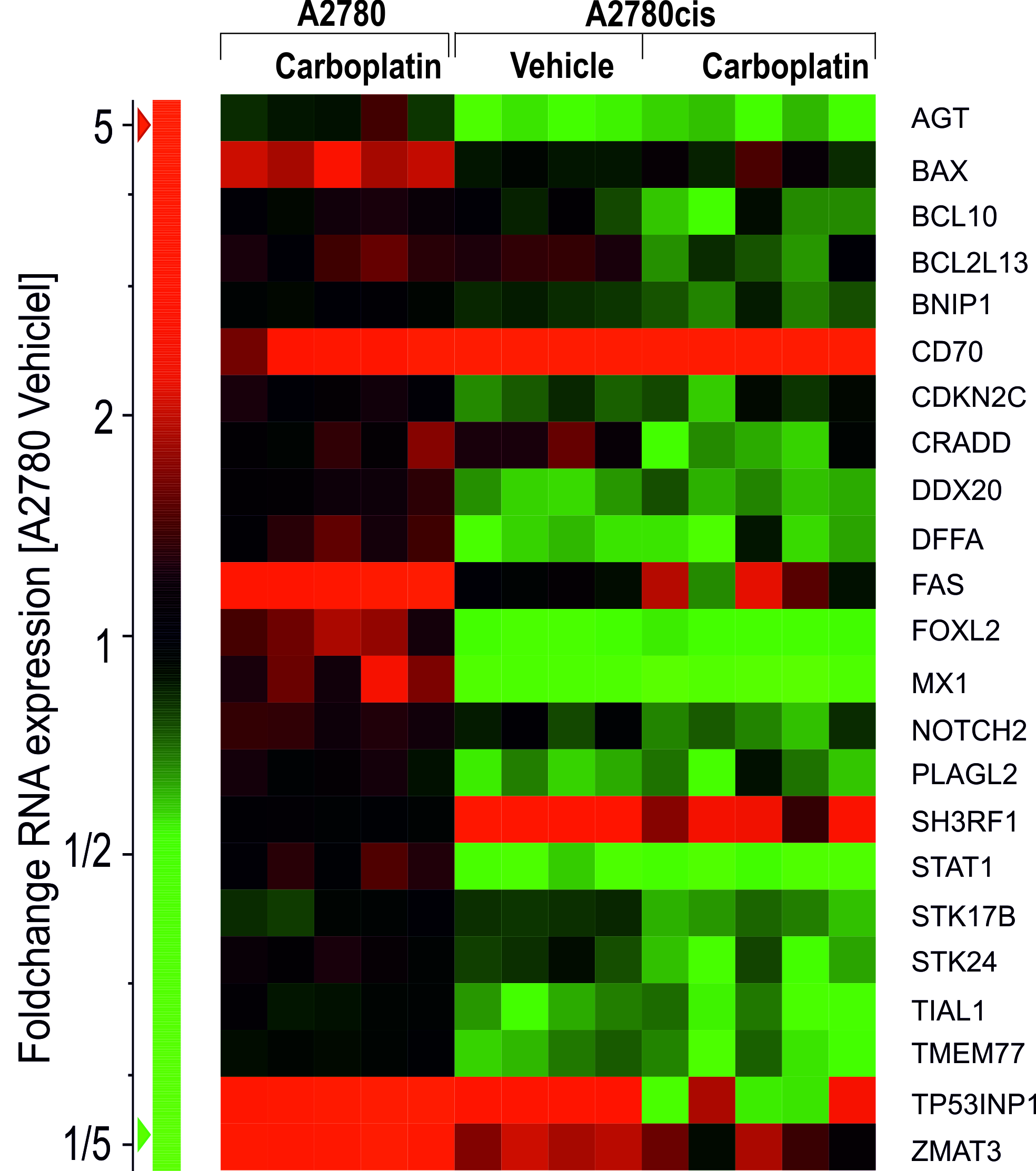

Supplement: Supplementary file 2 [file cam40004-0253-sd2.docx]

*
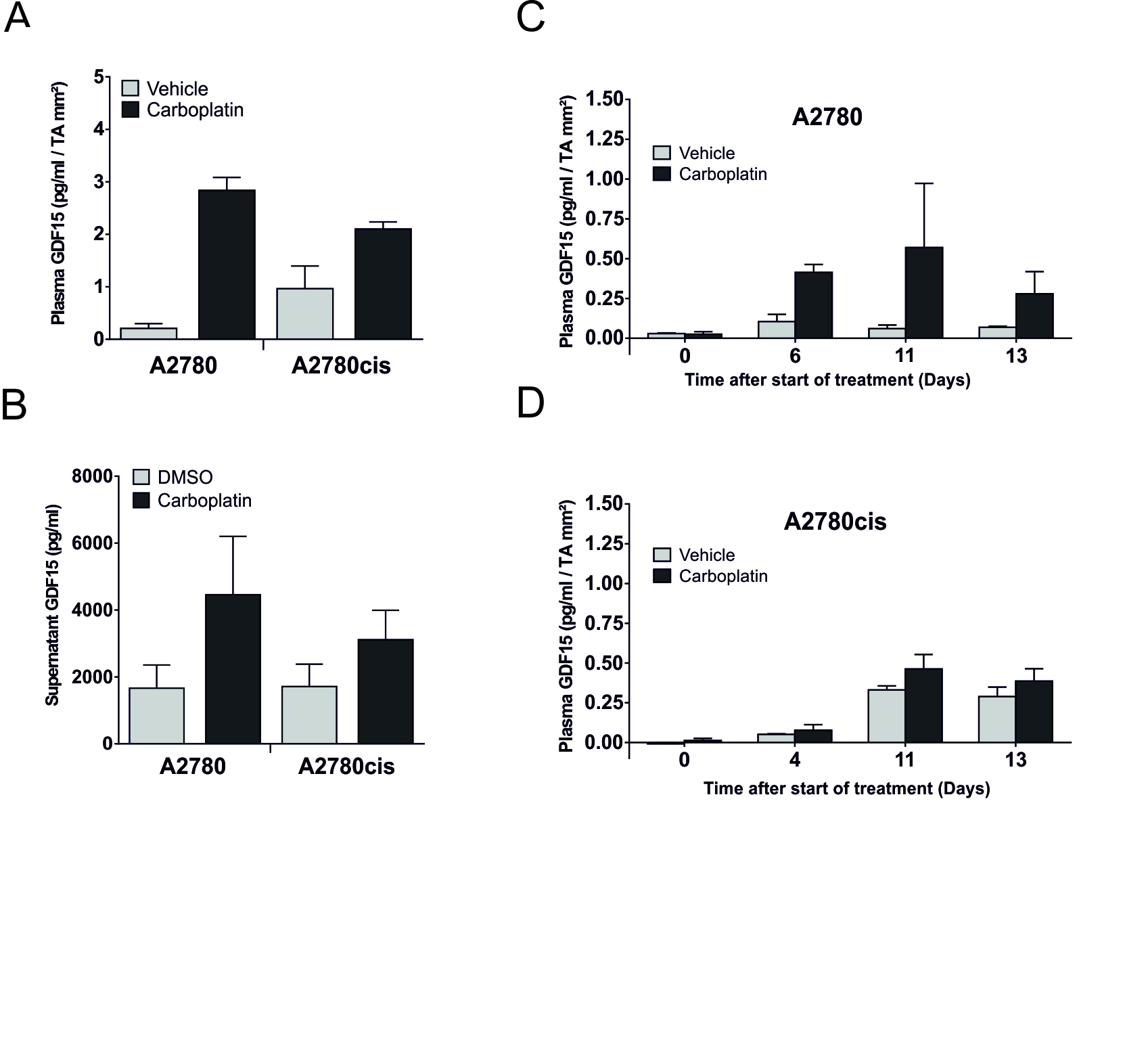
*

Supplement: Supplementary file 3 [file cam40004-0253-sd3.docx]

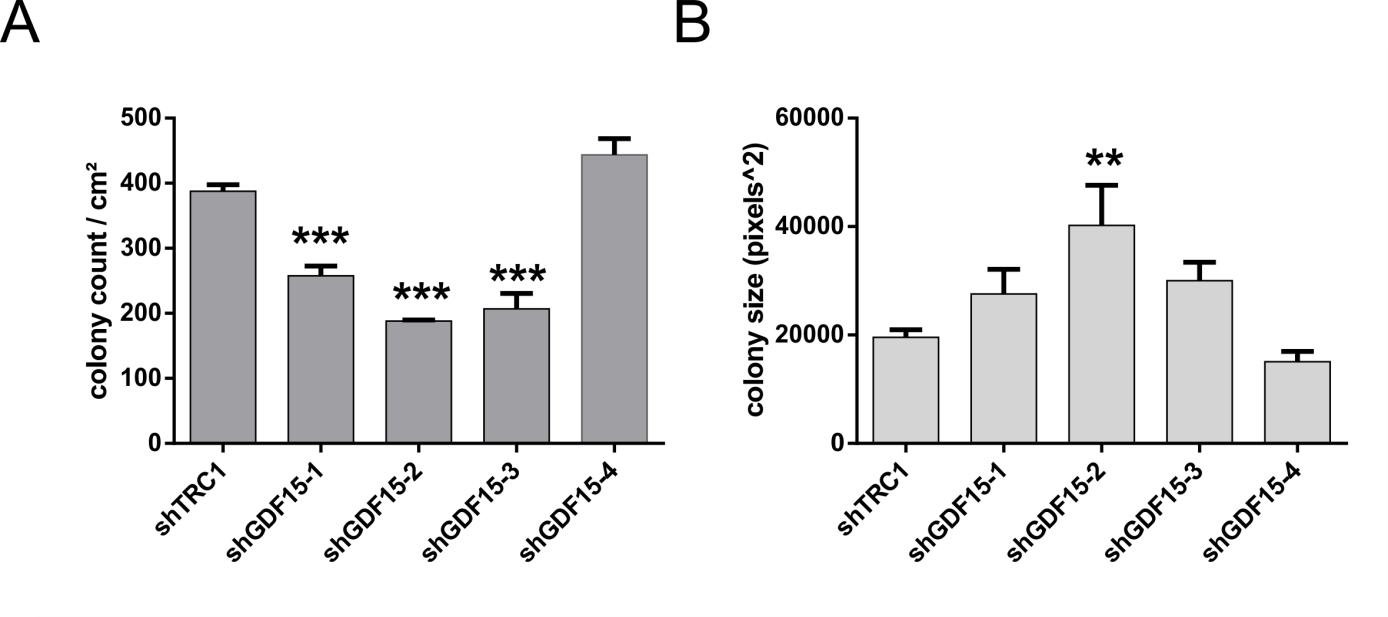

Supplement: Supplementary file 4 [file cam40004-0253-sd4.docx]

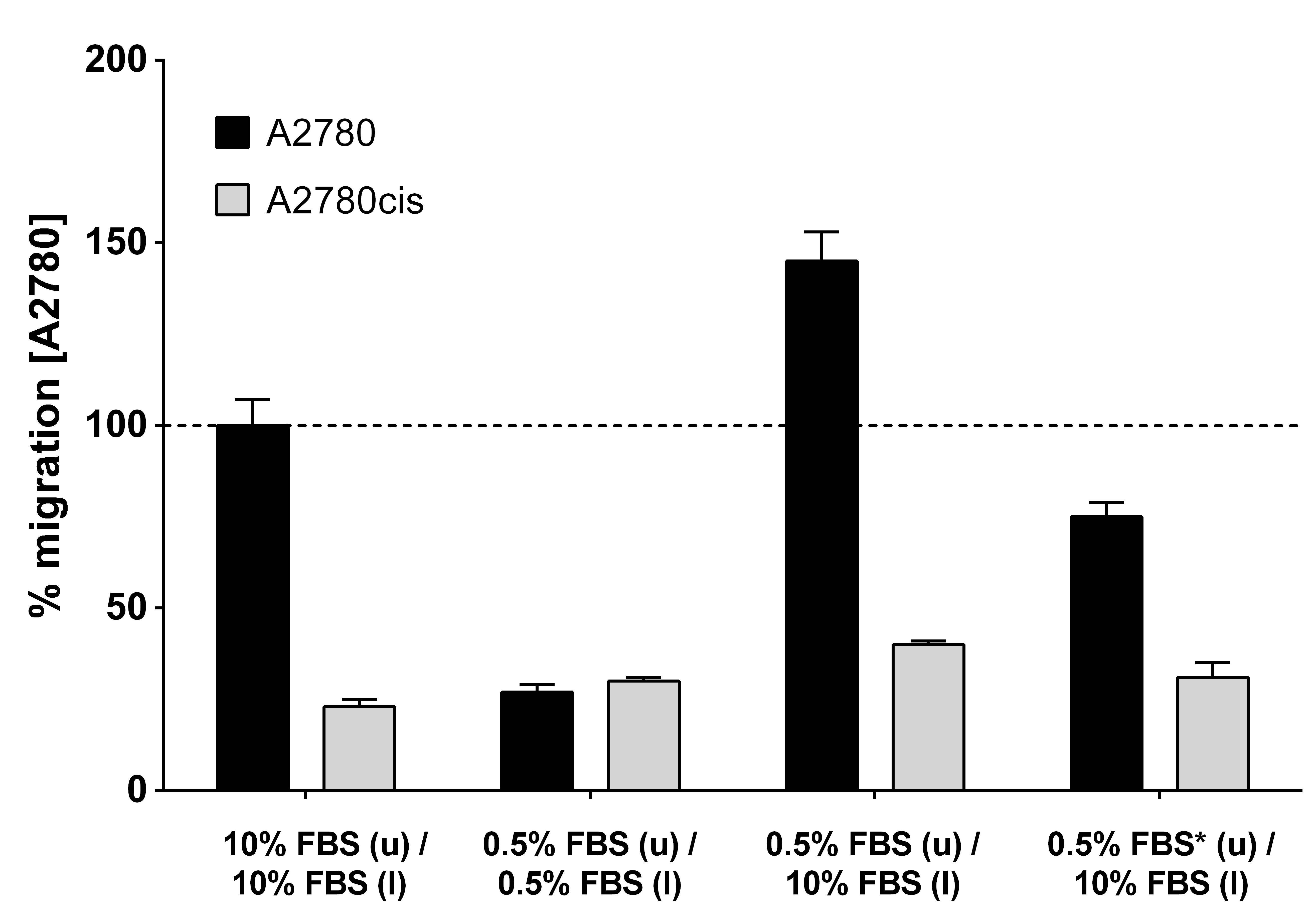

Supplement: Supplementary file 5 [file cam40004-0253-sd5.docx]

*
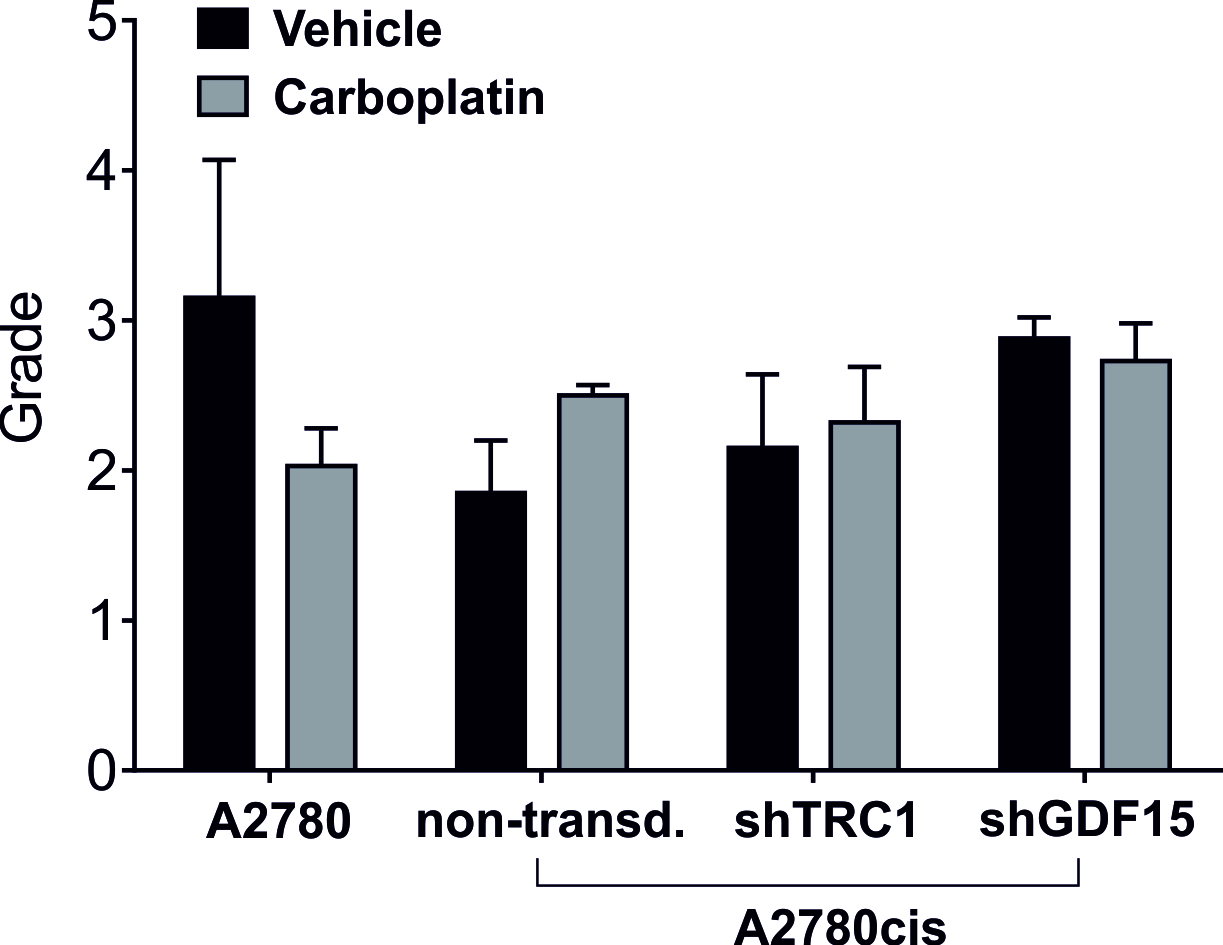
*

Supplement: Supplementary file 6 [file cam40004-0253-sd6.docx]
