# Supplementary material for: Knockdown of platinum-induced growth differentiation factor 15 abrogates p27-mediated tumor growth delay in the chemoresistant ovarian cancer model A2780cis
Source: Cancer Med. 2014 Dec 10;4(2):253–67. doi: 10.1002/cam4.354 (PMC4329009; doi:10.1002/cam4.354)
Supplement: Supplementary file 7 [file cam40004-0253-sd7.docx]

**Supplementary Table S1**

| **A2780cis-** | **% crystal violet staining** | |
| --- | --- | --- |
|  | **mean** | **SD** |
| shTRC1 | 100.0 | 2.1 |
| shGDF15 - 1 | 117.5 | 1.4 |
| shGDF15 - 2 | 116.8 | 2.3 |
| shGDF15 - 3 | 104.3 | 0.5 |
| shGDF15 - 4 | 118.3 | 11.2 |
