# Supplementary material for: Knockdown of platinum-induced growth differentiation factor 15 abrogates p27-mediated tumor growth delay in the chemoresistant ovarian cancer model A2780cis
Source: Cancer Med. 2014 Dec 10;4(2):253–67. doi: 10.1002/cam4.354 (PMC4329009; doi:10.1002/cam4.354)
Supplement: Supplementary file 8 [file cam40004-0253-sd8.docx]

**Supplementary Table S2**

| **xenogenic tumor** | **treatment** | **days of growth until TA ≥ 150mm²** | **Lower 95% Confidence Limit** | **Upper 95% Confidence Limit** | **% growth inhibition to Vehicle** |
| --- | --- | --- | --- | --- | --- |
| A2780 | Vehicle | 8 | 8 | 9 | - |
|  | Carboplatin | 11 | 14 | 26 | 37.5 |
| A2780cis | Vehicle | 15 | 12 | 19 | - |
|  | Carboplatin | 20 | 18 | 22 | 33.33 |
| shTRC1-cis | Vehicle | 15 | 13 | 18 | - |
|  | Carboplatin | 20 | 13 | 23 | 33.33 |
| shGDF15-cis | Vehicle | 12 | 11 | 15 | - |
|  | Carboplatin | 18 | 11 | 20 | 50 |
